# Coordinated cellular responses track cognitive outcomes in Alzheimer’s disease

**DOI:** 10.21203/rs.3.rs-11296978/v1

**Published:** 2026-10-20

**Authors:** Panos Roussos, Pengfei Dong, Xuan Cao, Seon Kinrot, Clara Casey, Aram Hong, Marcela Alvia, Zhiping Shao, Joana Krause-Massaguer, Vahram Haroutunian, Gabriel Hoffman, Jaroslav Bendl, Donghoon Lee, John Fullard

## Abstract

Individuals with comparable Alzheimer’s disease (AD) pathology can differ markedly in cognition, but the molecular nature of this heterogeneity remains unclear. Here we profiled paired gene expression and chromatin accessibility in 1.42 million nuclei from three cortical regions of 247 donors. A pathology-supervised transcriptomic score, supported by paired chromatin measurements, resolved cellular disease state differences within samples and between individuals. After adjustment for measured pathology, cellular responses were concordant across major cell classes and cortical regions. Higher pathology-adjusted responses were associated with lower relative abundance of vulnerable excitatory and somatostatin-expressing inhibitory neurons and poorer cognitive outcomes. Pathology-associated, pathology-adjusted, and within-sample cellular-state differences converged on overlapping molecular programs. Unsupervised analysis identified reproducible donor-associated transcriptional programs, including a proteostasis and cellular-stress program associated with higher pathology-adjusted responses, contrasting a lipid-metabolic and biosynthetic program showing the opposite relationship across regions and cohorts. Together, these findings identify a coordinated molecular phenotype associated with differences in neuronal composition and cognition among individuals with comparable measured neuropathology.

